# Chronic Cadmium Exposure Alters Cardiac Matrix Metalloproteinases in the Heart of Sprague-Dawley Rat

**DOI:** 10.3389/fphar.2021.663048

**Published:** 2021-08-10

**Authors:** Sandra Concepcion Das, Kavitha Varadharajan, Muralitharan Shanmugakonar, Hamda A. Al-Naemi

**Affiliations:** ^1^ Laboratory Animal Research Center, Qatar University, Doha, Qatar; ^2^ Department of Biological and Environmental Sciences, Qatar University, Doha, Qatar

**Keywords:** cadmium, cardiac, inflammatory cytokines, MMP, TIMP

## Abstract

The aim of this study was to evaluate the role of chronic cadmium exposure in modulating cardiac matrix metalloproteinases (MMPs) in the heart of rats. Adult male Sprague-Dawley rats were exposed to 15 ppm CdCl_2_ in drinking water for 10 weeks followed by withdrawal of cadmium treatment for 4 weeks. Following the completion of the treatment, gene expression of inflammatory mediators (*IL-1β*, IL-6, IL-10, TNF*-*α and NF-κB), protein expression of MMP-2, MMP-9 and their respective inhibitors- TIMP-1 and TIMP-2, and gelatinolytic activity of MMP-2 and MMP-9 were determined. At the protein level, cadmium incites a differential effect on the expression and activity of gelatinases and their endogenous inhibitors in an exposure-dependent manner. Results also show that the administered cadmium dose elicits an inflammatory response until week 10 that slightly diminishes after 4 weeks. This study provides evidence of cadmium-induced imbalance in the MMP-TIMP system in the cardiac tissue. This imbalance may be mediated by cadmium-induced inflammation that could contribute to various cardiovascular pathologies.

## Introduction

Since the discovery of cadmium in 1817 (National Center for Biotechnology Information, 2020), this element and its compounds has gained increasing industrial utility. However, despite its importance in industrial applications, cadmium has been identified as one among 126 priority pollutants (US Environmental Protection Agency (EPA), 2014), a Class I carcinogen (International Agency for Research on Cancer, 2012) and is considered a pollutant of the environment and toxicant for health (Nordic Council of Ministers, 2003; Faroon et al., 2012). The use of cadmium has been limited by international legislation. However, individuals may experience occupational or environmental exposure to cadmium through different route that might have implications on public health. For the non-smoking population, the major source of exposure is by ingestion of cadmium contaminated food and water (Faroon et al., 2012). As a subsequence of cadmium uptake into the organism, cadmium is widely transported and distributed to various tissues via the blood circulation. Hence, the ensuing toxic impact of cadmium causes adverse impacts on the various organs of the body.

Even at low doses, cadmium has been reported to impart its toxic effects on other organ systems including the nervous system (Méndez-Armenta and Ríos, 2007; Branca et al., 2018), immune system (Kulas et al., 2019; Mirkov et al., 2021), reproductive system (Thompson and Bannigan, 2008; de Angelis et al., 2017) and the cardiovascular system (Tinkov et al., 2018). With regards to the cardiovascular system, there is an increasing amount of epidemiological evidence showing that cadmium has an impact on cardiovascular health (Lee et al., 2011; Fagerberg et al., 2015; Deering et al., 2018; Gao et al., 2018; Noor et al., 2018). Studies have also reported the deposition and accumulation of cadmium in the heart and arterial tissue in both humans (Egger et al., 2019) as well as animal models (Erdem et al., 2016; Young et al., 2019). Histopathological studies of cardiovascular tissues after cadmium exposure have reported alteration of tissue structure and integrity, fibrosis and depletion of collagen fiber (Veličkov et al., 2013; Sangartit et al., 2014; Saleh and Awadin, 2017; Bhattacharjee et al., 2019). It was reported that the heart tissue accumulated the lowest amount of cadmium after the liver and kidney (Erdem et al., 2016). However, from the previously cited studies, it can be inferred that even at low concentrations of cadmium, there are biochemical and molecular alterations in the heart. It is not well understood yet how cadmium imposes varying degrees of effects on the heart tissue that leads to cardiovascular injury. From a mechanistic perspective, the detrimental remodeling reported in cardiac tissue after cadmium exposure may be attributed to the dysfunction of the expression or activity of matrix metalloproteinases and their corresponding inhibitors.

In the context of cardiovascular dysfunction, a hallmark of chronic cadmium exposure is the induction of fibrosis (Bhattacharjee et al., 2019). Critical to the process of fibrosis is the balance between matrix metalloproteinases (MMPs) and tissue inhibitors of metalloproteinases (TIMPs) (Liu et al., 2006; Azevedo et al., 2014). MMPs orchestrate and modulate several facets of inflammation such as integrity of tissue barriers, recruitment of inflammatory cells and release of cytokines (Szabo et al., 2004; Nissinen and Kähäri, 2014). From the numerous MMPs characterized and identified, gelatinases (MMP-2 and MMP-9) play a role in remodeling of the cardiac infrastructure. They have been reported to play a role in inflammation-induced imbalance of extracellular matrix turnover in the heart (Pytliak et al., 2017). It has been demonstrated that non-toxic cadmium stimulates MMP-9 and MMP-2 expression in the cardiovascular system inducing vascular inflammation and promoting atherosclerosis (Knoflach et al., 2011). The mechanism by which cadmium-induced inflammation mediates the imbalance of MMPs leading to cardiac fibrosis has not been completely elucidated.

Therefore, this study aims to evaluate the role of cadmium in modulating cardiac MMPs in the heart of rats after cadmium exposure.

## Materials and Methods

### Study Design

All experimental procedures performed are in accordance with the National Institute of Health Guide for the Care and Use of Laboratory Animals (NIH Publications No.8023, revised 1978) and were approved by the Institutional Animal Care and Use Committee of Qatar University (Approval # QU-IACUC 038/2017). The samples obtained in this study were acquired from the animals employed in a previous study from our research group (Al-Naemi and Das, 2020).

**FIGURE 1:**
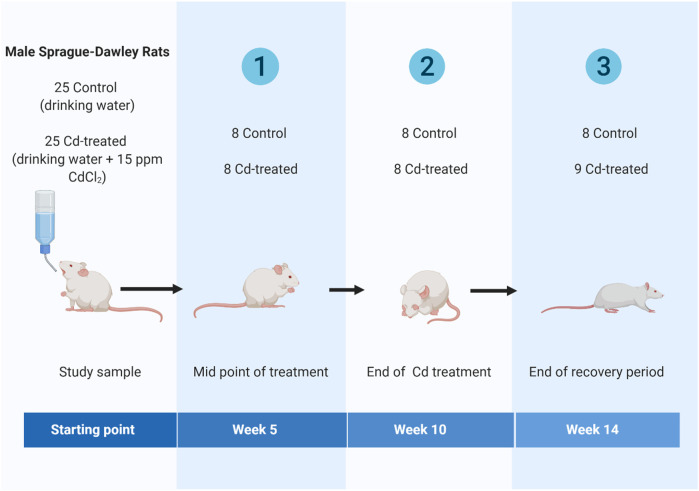
Scheme illustrating the timeline of the study design(Created using Biorender).

Briefly, adult (8 weeks old) male Sprague-Dawley rats were obtained from Laboratory Animal Research Center, Qatar University and housed in individually ventilated cages (IVC) under standard conditions (room temperature: 18–22°C, relative humidity: 40–60% and 12 h light: 12 h dark cycle). Animals were randomly assigned to one of two regimes for 14 weeks- 1) control group or 2) cadmium treatment (Cd-treated) (Figure 1). The control group received drinking water for 14 weeks. The Cd-treated group received 15 ppm (15 mg/kg body weight) as CdCl_2_ (BDH, Qatar) in drinking water for 10 weeks followed by 4 weeks of normal drinking water. The administered cadmium exposure dose was selected based on previous literature reporting that a dose of 15 ppm of CdCl_2_ is a non-carcinogenic dose (Alvarez et al., 2004) leading to a circulating serum concentration of 5 ppb-the toxic limit in humans by the WHO (Ferramola et al., 2011; Ferramola et al., 2012). The calculated human equivalent dose (HED) (Reagan-Shaw et al., 2008; Ghosh and Indra, 2018) for the selected animal dose (15 mg/kg body weight/day) is 2.4 mg/kg which is similar to the reported intake (2.86–11.71 mg/kg body weight/day) in epidemiological studies (Bernhoft, 2013; Chunhabundit, 2016).

Animals received *ad libitum* access to standard rodent chow and drinking water during the experiment. Animals were sacrificed at weeks 5, 10 and 14 under anaesthesia with sodium thiopentone (40 mg/kg body weight, intraperitoneally). Heart tissue was dissected, cleaned from residual blood, immersed in liquid nitrogen, and preserved in the tissue archive at −80°C until further analysis.

### Preparation of Tissue Homogenates

Frozen heart samples were weighed to approximately 20–30 mg and homogenized by sonication at 40% amplitude in pulses of 5 s. Heart tissue was homogenized in radioimmunoprecipitation assay (RIPA) cell lysis buffer to extract total proteins in combination with cocktail protease inhibitor and TRIzol reagent to extract RNA.

### Protein Extraction and Quantification

Sonicated heart tissue samples were lysed for crude protein by incubating in RIPA cell lysis buffer (ThermoFisher-Scientific, United States; 78440) containing cocktail protease inhibitor (ThermoFisher-Scientific, United States; 87786). Protein extraction for MMP activity assay included EDTA in the cell lysis buffer. Cellular debris was pelleted by centrifugation for 10,000 rpm, 10 min at 4°C. The supernatant containing total crude protein concentration was estimated using Quick Start™ Bradford Protein assay (Bio-rad, United States; 5000205) as per instructions in the user’s manual provided (Bradford, 1976).

### Western Blot Analysis

The protein expression of gelatinases i.e., MMP-2 & MMP-9 (Sigma-Aldrich, United States; MAB3308, MAB3309) and their respective inhibitors-TIMP-1 & TIMP-2 (Sigma-Aldrich, United States; MAB3310; Abcam, United States; ab61224) in response to cadmium treatment were analyzed by Western blotting. Equal amounts of crude protein was loaded and resolved by reducing SDS-PAGE. Separated proteins were electroblotted to polyvinylidene difluoride (PVDF) membrane (GE Healthcare Limited, Buckinghamshire). Electroblots were washed with TBST (20 mM Tris-buffered saline and 0.1% Tween-20), blocked and probed with primary antibodies for the targets overnight at 4°C. Detection was done using compatible secondary HRP-conjugated antibodies (Abcam, United States; ab205718, ab205719) and incubated in ECL solution for a minute. Immunoreactive bands were visualized using ECL (ThermoFisher-Scientific, United States; 32209) under SynGene Gel documentation system. Protein expressions was evaluated by densitometric analysis using Image studio Lite (Ver 5.2) and normalized against the corresponding expression of GAPDH. Analysis was presented as a fold change compared to the respective controls.

### Zymography

Gelatinolytic activity of MMP-9 and MMP-2 was determined by gelatin zymography. Equal amounts of crude protein (40 µg) were mixed with sample loading buffer [0.25 M Tris (pH 6.8), 30% glycerol, 1% SDS and 0.02% bromophenol blue] and were loaded into a 9% acrylamide:bis-arcylamide gel containing 0.1% gelatin (Sigma-Aldrich, United States; G9391) as substrate. Non-reducing electrophoresis was done. After electrophoresis, the gel was washed twice in renaturing buffer containing 2.5% Triton X-100 at 37°C and incubated in developing buffer [50 mM Tris-HCl (pH 7.8), 0.2 M NaCl, 5 mM CaCl_2_ and 0.02% Triton X-100] at 37°C until a clear lysis zone is observed. The gel was quick stained in 0.10% Coomassie brilliant blue R250 for 5 min and destained till contrast between the lysis bands and blue gel background was visible. The zymographs were documented using the SynGel Documentation system. The proteolytic activity was analyzed using ImageJ and presented as a fold change compared to the control.

### RNA Extraction and Quantification

Total RNA was isolated from frozen heart samples using TRIzol reagent (Invitrogen, United States) according to the manufacturer’s instructions as previously described (Rio et al., 2010). Total RNA concentration and quality was determined using Nanophotometer (IMPLEN) at 260/280 nm as described previously (Koetsier and Cantor, 2019).

### Gene Expression Analysis

The cardiac mRNA expression levels of specific inflammatory mediators in response to cadmium treatment was analyzed using real-time polymerase chain reaction (RT-PCR). Extracted total RNA was reverse transcribed to cDNA using High-Capacity cDNA reverse transcriptase kit (Applied Biosystems, Lithuania; 4374966) as per instructed in user’s manual. Total cDNA was diluted by 4-fold in RNase-free water. The gene expression assay was performed using Quant Studio 6 Flex Real Time PCR system (Applied Biosystems). Amplification plots were obtained for each target using specific primers for IL-1β, IL-6, IL-10, TNF*-*α, NF-κB and GAPDH individually (TaqMan Gene expression assay, ThermoFisher-Scientific, United States; Rn00580432_m1, Rn01483988_g1, Rn01410330_m1, Rn01475473_m1, Rn01525859_g1, Rn01775763_g1, respectively). Quantification was done by the ΔΔC_t_ method wherein results were compared against both calibrator (control group) and normalizer (endogenous, GAPDH) to obtain the ΔΔC_t_ value (Pfaffl, 2004). This value was used to determine the fold difference in expression.

### Statistical Analysis

The results were presented as mean ± S.E.M and analyzed by two-way ANOVA followed by Tukey’s *post-hoc* test using GraphPad Prism (ver.8.4) for Windows (GraphPad Software, San Diego, California, United States). *p*-value < 0.05 was considered as statistically significant.

## Results

### Level of Protein Expression of Matrix Metalloproteinases in Heart Tissue

**FIGURE 2:**
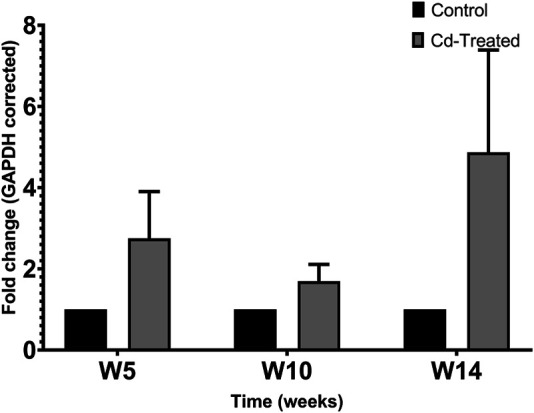
Effect of Cd-treatment on expression of MMP-2 in heart tissue of male Sprague-Dawley rats. Protein expression data were normalized against the expression of GAPDH and expressed as mean ± S.E.M of fold-changes compared to the control (*n* = 8).

To evaluate the level of expression of MMPs, 40 µg of crude protein was separated in 9% SDS-PAGE. Protein expression was evaluated by probing electroblots specific for MMP-2 and MMP-9. By western blotting, only MMP-2 was detectable at approximately 64 kDa. It was observed that MMP-2 increased by more than 2-fold at week 5, slightly decreased at week 10 and reaches a maximum fold change of more than 4-fold at week 14 (Figure 2). In comparison to the respective control groups, the changes were not statistically significant at each time point.

### Level of Protein Expression of TIMPs in Heart Tissue

**FIGURE 3:**
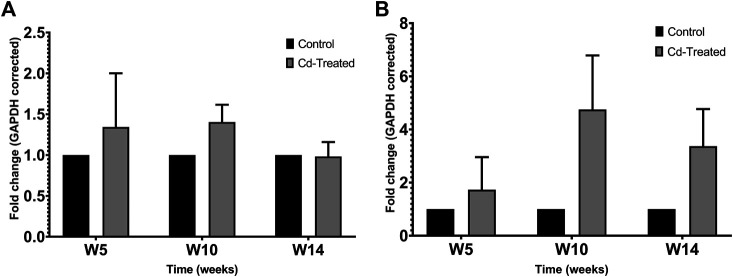
Effect of Cd-treatment on expression of TIMP in heart tissue of male Sprague-Dawley rats. Protein expression data of **(A)** TIMP-1 and **(B)**TIMP-2 were normalized against the expression of GAPDH and expressed as mean ± S.E.M of fold-changes compared to the control (*n* = 8).

Protein expression of TIMPs was assessed by separating 50 µg of crude protein in 12% SDS-PAGE and probing immunoblots against TIMP-1 and TIMP-2. The expression of TIMP-1 showed limited changes at the level of the protein in comparison to the control group (Figure 3A). Less than 1.5-fold change was observed at week 5, which was sustained at the same level till week 10 and mildly decreases to be similar to the level of expression of the control group at week 14. A contrasting pattern is observed for the expression of TIMP-2 (Figure 3B). The expression of TIMP-2 increased by 2-fold at week 5 reaching a maximum fold change of more than 4 times at week 10 and decreases by about 3-fold at week 14. The changes shown in Figure 3 were not statistically significant.

### Gelatinolytic Activity of Matrix Metalloproteinases in Heart Tissue

**FIGURE 4:**
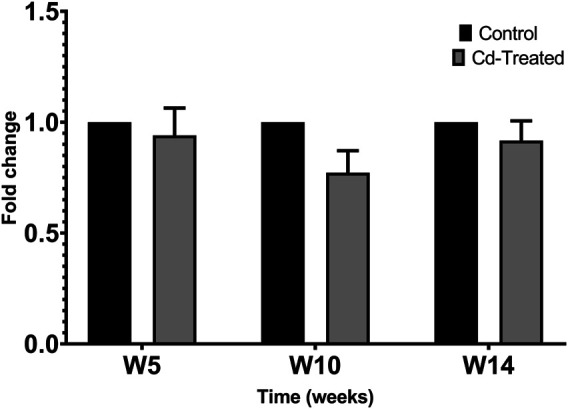
Gelatinolytic activity of pro-MMP-2 after 24 h. Data is expressed as mean ± S.E.M of fold-changes compared to the control (*n* = 8).

Activity of MMPs were evaluated by gelatine zymography indicated by visualization of bands of lysis corresponding to the molecular weight of MMP-2 and MMP-9. After 24 h incubation, activity corresponding to the proenzyme form of MMP-2 at 72 kDa was observed. Densitometric analysis of the detected area of lysis shows that the activity of pro-MMP-2 does not increase by more than 1-fold at all time points in comparison to their respective control group (Figure 4).

**FIGURE 5:**
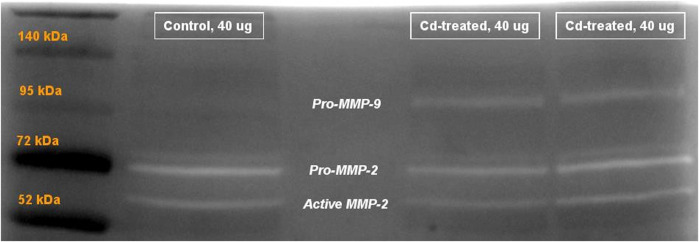
Representative image showing gelatinolytic activity of MMP after 60+ h incubation. Labels show the activity of pro-MMP-9 at 95 kDa, pro-MMP-2 at 72 kDa and active MMP-2 at approximately 65 kDa. The activity of active MMP-9 (82 kDa) was not visible after 60+ h.

**FIGURE 6:**
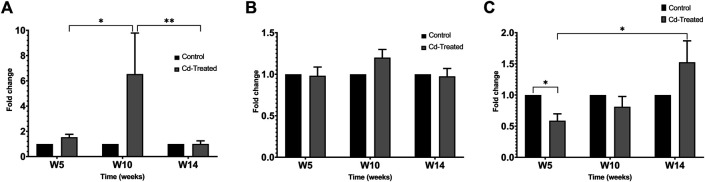
Gelatinolytic activity of MMP-2 and MMP-9 after incubation **(A)** pro-MMP-9 after 60+ h **(B)** pro-MMP-2 after 60+ h and **(C)** active MMP-2 after 60+ h is expressed as mean ± S.E.M of fold-changes compared to the control (*n* = 8). **p < 0.01,*p < 0.05.

On extension of the incubation period to more than 60 h, the activity of proenzyme form of MMP-9 and both proenzyme and active enzyme form of MMP-2 was detected (Figure 5). Quantification of the activity for each of the enzyme forms was done by densitometric analysis. At week 5, the activity of pro-MMP-9 increased by 2-fold to reach a maximum increase of more than 6-fold at week 10 and decreased to 1-fold at week 14 in comparison to their respective control samples (Figure 6A). The increase in gelatinolytic activity from week 5 to week 10 was found to be statistically significant (*p* < 0.05). Also, the decrease in activity from week 10 to week 14 was found to be statistically significant (*p* < 0.01). In regard to proenzyme MMP-2 (Figure 6B), the activity of Cd-treated group at week 5 and week 14 shows similar activity to their respective control group. However, at week 10, a slight increase in activity is observed in the Cd-treated group. With respect to the activity of active form of MMP-2 (Figure 6C), there is a 0.5-fold decrease at week 5 that slightly increases at week 10 and reaches a maximum of 1.5-fold increase at week 14. The fold change observed at week 5 was found to be statistically significant in comparison to the control group while the differences observed between week 5 and week 14 was also found to be statistically significant (*p* < 0.05).

### Level of Gene Expression of Inflammatory Mediators in Heart Tissue

**FIGURE 7:**
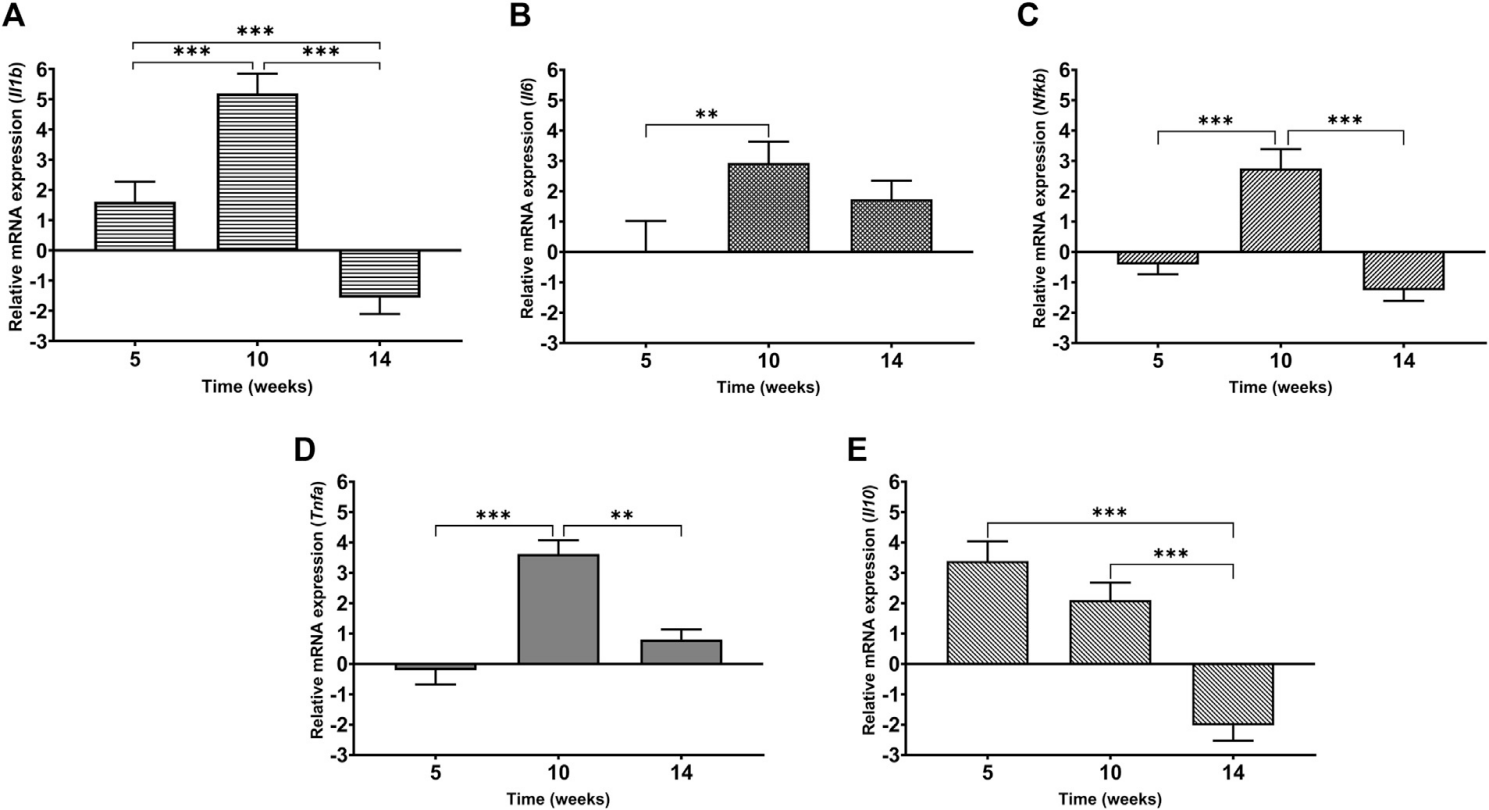
Levels of mRNA expression of inflammatory mediators in heart tissue of Cd-treated male Sprague-Dawley rats using real time-PCR **(A)** IL-1β **(B)** IL-6 **(C)** NF-κB **(D)** TNF-α **(E)** IL-10. Expression data are represented as mean ± S.E.M of relative mRNA expression compared to the control (*n* = 8). ***p < 0.001, **p < 0.01.

In order to evaluate the inflammatory status of the heart under cadmium treatment over time, the levels of mRNA expression of IL-1β*,* IL-6, IL-10, NF-κB and TNF-α were assessed and represented as a fold change (Figure 7 (A-E)). At week 5, it is observed that IL-1β and IL-10 are upregulated by 1.5-fold and more than 3-fold, respectively. In the same time point, both NF-κB and TNF*-α* are slightly downregulated by less than 1-fold (Figures 6D, 7C). In contrast to week 5, it is observed that at week 10, the pro-inflammatory mediators- IL-6, IL-1β, NF-κB and TNF-α are significantly upregulated (Figures 7A–D). Following through to week 14, it was observed that three out of the five mediators i.e., IL-1β, IL-10 and NF-κB have significantly downregulated while the others (IL-6 and TNF-α) have a sustained expression by 2-fold and 1-fold, respectively. It must be noted that maximum fold change was observed at week 10 for all mediators except anti-inflammatory cytokine, IL*-*10.

## Discussion

Cadmium is an environmental priority pollutant with reported associated repercussions on various organ systems including the cardiovascular system. Recent studies have reported the relevance of cadmium as a novel and independent risk factor for pathologies of the cardiovascular system (Messner and Bernhard, 2010; Tellez-Plaza et al., 2013; Solenkova et al., 2014; Santos-Gallego and Jialal, 2016; Lamas et al., 2021). A common pathology reported in the previous studies is loss of structural integrity of the cardiac tissue observed by formation of irregular branching pattern, complexed matrix network, fibrosis and focal areas of necrosis (Sangartit et al., 2014; Saleh and Awadin, 2017; Bhattacharjee et al., 2019). These studies demonstrated that cadmium mediates the tissue damage of the cardiovascular system by inducing oxidative stress. However, another pathway is the simultaneous implication of inflammation with oxidative stress during cadmium exposure (Liu et al., 2015; Kukongviriyapan et al., 2016; Tucovic et al., 2018; Koopsamy Naidoo et al., 2019). Previous work has linked cadmium-induced oxidative stress with cardiovascular pathologies in both humans and animals (Lee et al., 2006; Ferramola et al., 2011; Ferramola et al., 2012; Liang et al., 2019). A prior study using similar experimental conditions of 15 ppm for 8 weeks has reported the histopathological alterations in the heart of male Wistar rats in correlation with circulating inflammatory markers (Yazihan et al., 2011a). Another group using the same dose and duration reported ultrastructural degenerative changes in the cardiac tissue (Ozturk et al., 2009). The study by Saleh and Awadin (2017) observed fibrosis of the heart tissue of male albino rats under similar dose and duration. Collectively these data showed evidence of histopathological changes to the cardiac tissue after cadmium exposure. To our knowledge, there is limited available data on the level of MMP expression so the current study takes a focused molecular approach to understand the effect of chronic cadmium exposure that might contribute in the functionality of the cardiac MMPs.

Matrix metalloproteinases (MMPs) play a vital role in both extracellular matrix turnover and inflammatory response in normal physiology. A dysfunction in their expression and activity, specifically gelatinases has been widely implicated in various cardiovascular pathologies (Peterson, 2000; Spinale, 2007; Wang and Khalil, 2018). The current study evaluated both the expression as well the activity of MMP-2 and MMP-9 in the heart tissue under cadmium stress whereas majority of published studies exploring the impact of heavy metal exposure on MMP was evaluated by gelatinolytic zymography. It is worth highlighting that although the protein expression of MMP-9 may be undetectable, this does not imply the absence or lack of activity of the protein as shown in this study. Despite the non-detectable MMP-9 protein by immunoblotting, enzymatic activity of MMP-9 was observed after 60+ h of incubation only. This supports the idea that the protein expression levels of MMP-9 may be very low under this experimental setup however, the protein still retains its activity. MMP-9 is an inducible protein that is synthesized by inflammatory cells when stimulated by the release of IL-1β, IL-6 or TNF-α (Rutschow et al., 2006; Hu et al., 2007). Furthermore, an *in vitro* study in cardiac fibroblasts demonstrated that IL-1β and TNF-α increased the total activity of MMP-2 and MMP-9 suggesting that the increase in activity may be partly attributed to the increase in the transcription of gelatinases (Siwik et al., 2000). Similarly, in the present study, the results corresponding to the increase in activity of latent MMP-2 and MMP-9 at week 10 also corresponds in parallel to the upregulation in the gene expression of pro-inflammatory cytokines. This substantiates the hypothesis that cadmium incites an inflammatory response that stimulates the activity of pro-MMPs in the cardiac tissue. With regard to MMP-2 activity, the pro-enzyme form seems to have minimal variation over time. Contrastingly, the activity of active form of MMP-2 was observed to gradually increase over time in the cadmium treated group. These observations suggest that cadmium interferes with the activity of MMP-2 such that even though the pro-enzyme form may be available, there may be an interference in the activation of the pro-enzyme form to active form due to the cadmium treatment. Taken together, it seems that chronic dose of cadmium exposure modulates both expression and activity of MMP-2 and MMP-9 in the heart. However, differences in expression and activity of gelatinases following cadmium exposure may be attributed to the dose, duration, route of exposure and biological set up. Further research into MMP-9 is required to confirm modulation in protein expression in the heart.

One of the strategies to regulate and modulate the expression of MMPs is by means of TIMPs. The inhibitory relationship of the TIMPs on their preferential MMPs was clearly reflected in the protein expression levels of TIMP-1 and TIMP-2. Furthermore, the expression of TIMP-2 can be inversely correlated with the expression of MMP-2 wherein an elevation in the expression of TIMP-2 decreased the expression of MMP-2. These results demonstrate the effect of cadmium on the expression of TIMPs and hence the inhibition of MMPs. An *in vitro* study on cadmium treated U-937 cells showed that cadmium doses (1.0–50.0 µM) did not have any effect of TIMP-1 levels however there was an alteration in the MMP-9/TIMP-1 expression at the level of the gene albeit not statistically significant (Yaghooti et al., 2012). The study concluded that cadmium disrupts MMP-9/TIMP-1 balance to favour proteolysis. Consistent with previous studies (Fiévez et al., 2009; Yaghooti et al., 2012), cadmium exposure modulates MMPs expression and activity by disrupting the balance between MMP and TIMPs. Apart from these findings, it cannot be overlooked that MMPs are zinc-dependent endopeptidases and there also lies the possibility that cadmium may play a role in deactivating the gelatinases by mimicking the divalent zinc in these enzymes, also known as molecular mimicry (Bridges and Zalups, 2005; Chmielowska-Bąk et al., 2013). This remains to be confirmed by further research.

It is well-documented that IL-1β, IL-6 and TNF-α cause inflammation, therefore changes in gene expression of these cytokines are indicative of inflammation (Ptaschinski and Lukacs, 2018; Das and Al-Naemi, 2019). In the current study, the gene expression of targeted inflammatory mediators (IL-1β, IL-6, IL-10 and TNF-α) in the heart tissue of rats across three time points after cadmium treatment showed an exposure-dependent variation in their expression. After 5 weeks of cadmium exposure, cardiac tissue showed an upregulation of IL-1β and IL-10 and downregulation of NF-κB and TNF-α. At week 10, all the targets are upregulated while discontinuation of cadmium treatment for 4 weeks resulted in downregulation of the targets except IL-6 and TNF-α remaining upregulated. NF-κB is a vital player as a transcription factor in inflammation with a role in the regulation of several genes including cytokines (Kumar et al., 2004; Lawrence, 2009). Gordon et al. (2011) hypothesized that NF-κB regulates three genetic programs namely, hypertrophy, cytoprotection and chronic cytotoxicity brought on by prolonged inflammatory response. Chronic activation of the NF-κB signalling strongly drives the chemokine production by propagation of the pro-inflammatory cascade leading to a prolonged inflammatory state (Gordon et al., 2011; Ptaschinski and Lukacs, 2018). An *in vitro* study in ECV304 cells suggested that NF-κB was activated by the cadmium treatment via phosphorylation and degradation of the NF-κB inhibitor, IκBα (Lian et al., 2015). In the same study, cadmium consistently increased the transcriptional activity of NF-κB in a dose-dependent manner. Contradictory to previous studies our results showed that the expression of NF-κB in the cardiac tissue seems to vary by different folds. This suggests that the cumulative concentration of cadmium circulating within the system may not have reached a threshold to trigger the upregulation of NF-κB until at week 10. In the canonical signalling pathway of NF-κB, TNF-α is a widely studied ligand that activates NF-κB (Gordon et al., 2011).

TNF-α plays a pro-inflammatory role in local and systemic inflammation when secreted by activated macrophages in response to injury to amplify and prolong the inflammatory response (Tracey, 2002). A study reported a significant release of TNF-α in the heart of male rats treated at a dose of 5 mg/kg b.w. for 4 weeks (Nazimabashir et al., 2015). Another study reported that elevated levels of TNF-α in the heart of cadmium treated rats mediated a malfunction of the organ (Chen et al., 2003). In the current study, it was observed that TNF-α expression occurs in parallel to the expression of NF-κB until week 10 after which the expression of TNF-α opposes the expression of NF-κB. One of the main mechanisms of terminating the signal of NF-κB is by downregulation via a feedback loop involving TNF-α (Hayden and Ghosh, 2014). The downregulation of NF-κB and upregulation of TNF-α after recovery period (week 10–14) may be explained by this mechanism.

Extensive research exploring the impact of cadmium along the inflammation axis have reported the production and upregulation of IL-6, TNF-α and IL-1β both *in vivo* and *in vitro* (Djokic et al., 2015; Ninkov et al., 2015; Tucovic et al., 2018). In this study, the pro-inflammatory impact of the cadmium treatment was also observed as an upregulation in pro-inflammatory cytokines- IL-6 and IL-1β. Interestingly, the upregulation of anti-inflammatory cytokine, IL-10 was also observed till week 10 suggesting an activated mechanism to establish a balance in the inflammatory response. IL-6 is a key pro-inflammatory cytokine that mediates the transition from acute to chronic inflammation (Låg et al., 2010). For the current study, it must also be highlighted that after the recovery period (week 10–14), the sustained upregulation of IL-6 was observed. This suggests that an inflammatory signal remains stimulated despite the withdrawal of cadmium exposure for 4 weeks. However, it is also feasible that the circulating cadmium from the treatment or the presence of cadmium in the cardiac tissue (unpublished data) may in part play a role in provoking the inflammatory signal to still have an impact on the inflammatory status of the heart tissue. The results of this study are in agreement with other studies established in other biological model that reported differential effects of cadmium treatment on the expression of inflammatory cytokines *in vivo* and *in vitro* (Djokic et al., 2014; Djokic et al., 2015; Milnerowicz et al., 2015). Recent reviews have shown that the effects of cadmium may occur in an organ-specific manner by causing an imbalance in the pro-inflammatory/anti-inflammatory cascade as per the dose, exposure duration and biological model (Das and Al-Naemi, 2019; Hossein-Khannazer et al., 2020).

The present study did not include the role of chronic cadmium exposure on the kidneys and its relation to the described cardiac dysfunction has not been examined. On the other hand, several prior studies have reported that cadmium at the same dose has resulted in renal and cardiac injuries (Kacar Kocak et al., 2009; Yazihan et al., 2011b; Erdem et al., 2016). The authors reported hypertension associated with renal injury which may be mediated by oxidative stress, deteriorating bioelements like zinc, inducing apoptosis and inflammation. Our group previously reported the non-occurrence of hypertension with an overall depression in mean arterial pressure (Al-Naemi and Das, 2020). Considering the testing conditions that aimed to examine the direct effect of chronic cadmium exposure to the heart tissue while including an unsupplemented recovery period which has not been examined in previous studies, the involvement of inflammation and underlying mechanism needs further investigation. This would not only help in understanding the mechanisms underlying cardiovascular injury related to cadmium exposure but also in developing specific therapeutic strategies.

## Conclusion

The findings of this study provides considerable insight in understanding that despite the withdrawal of cadmium exposure, the impact of the exposure still persists in the heart. It must be noted that to the best of our knowledge, this study is the first to evaluate the impact of cadmium exposure on the heart after withdrawal of the treatment. This study shows that cadmium alters the expression and activity of gelatinases in the heart. Also, cadmium significantly stimulates an inflammatory response by the upregulation in the expression of pro-inflammatory cytokines that is still slightly sustained after withdrawal of the treatment. The sustained expression of cytokines can be attributed to the feedback regulation of MMPs to counter-regulate the inflammatory response and promote resolution (Fingleton, 2017). Alterations to the expression and activity of gelatinases impacts the heart ECM turnover to thereby modify the structure and function of the heart.

## Data Availability

The original contributions presented in the study are included in the article/supplementary materials, further inquiries can be directed to the corresponding author.
